# Endoscopic closure of a gastrocutaneous fistula reopening 18 years after gastrostomy removal using argon plasma coagulation and endoscopic hand suturing

**DOI:** 10.1055/a-2780-1150

**Published:** 2026-01-30

**Authors:** Ryota Yokoyama, Yutaka Okagawa, Atsushi Iwakubo, Norito Suzuki, Masahiro Hirakawa, Kohichi Takada

**Affiliations:** 1 92187 Division of Medical Oncology, Department of Internal Medicine, Sapporo Medical University School of Medicine, Sapporo, Japan

Reopening of a gastrocutaneous fistula (GCF) many years after gastrostomy tube removal is extremely uncommon 1 2 . In long-standing fistulas, the tract often becomes epithelialized with gastric mucosa, forming a rigid, mature channel. We report a rare case of GCF reopening 18 years after gastrostomy tube removal that was successfully closed using argon plasma coagulation (APC) followed by endoscopic hand suturing (EHS).

**Fig. 1:**
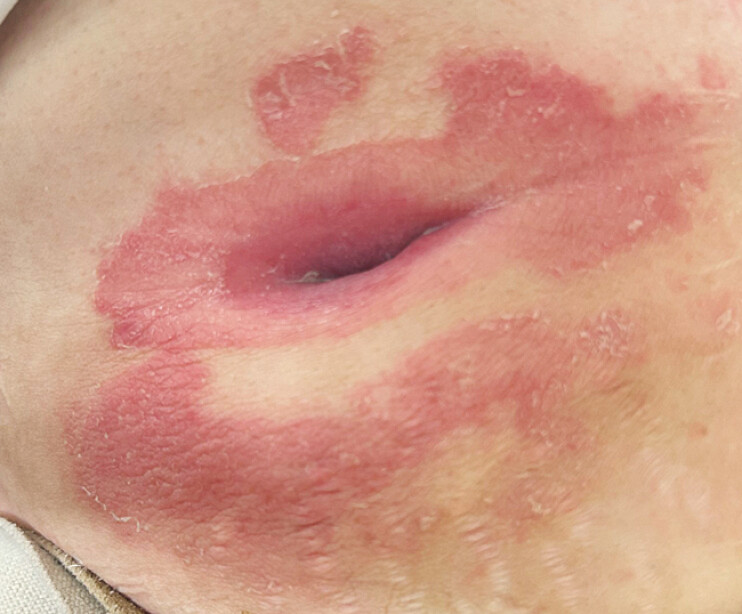
Abdominal skin erosion resulting from irritation by the leaked gastric fluid.

**Fig. 2:**
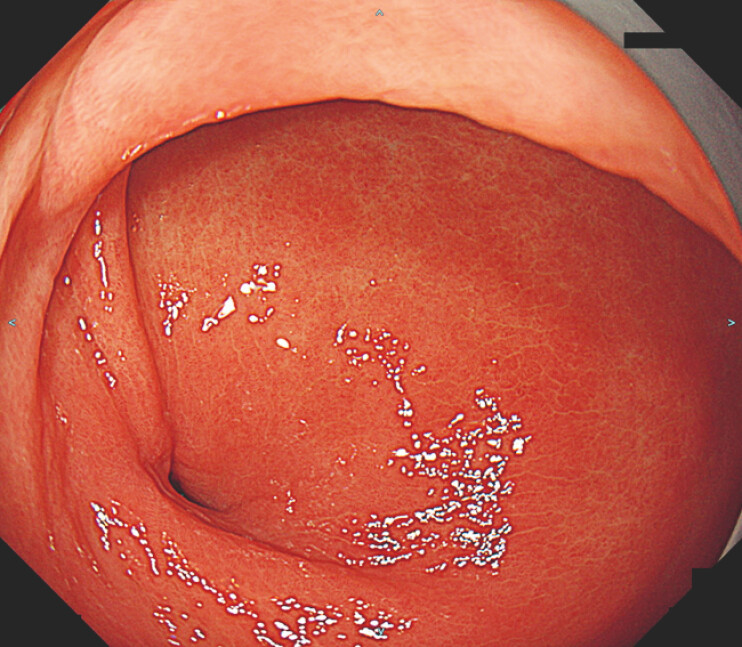
Esophagogastroduodenoscopy showed a fistulous orifice on the greater curvature of the antrum.

A 60-year-old woman with familial adenomatous polyposis had undergone total colectomy for colon cancer and surgical treatment for a ureteral desmoid tumor 23 years earlier. A percutaneous endoscopic gastrostomy was placed postoperatively for nutritional support and was removed once oral intake had recovered. The fistula closed spontaneously, and she remained asymptomatic for the following 18 years. Subsequently, gastric leakage developed from the previous gastrostomy site, leading to abdominal skin dermatitis caused by exposure to gastric fluid ( Fig. 1 ). Esophagogastroduodenoscopy (EGD) revealed a fistulous opening on the greater curvature of the antrum ( Fig. 2 ). Endoscopic clip closure was attempted; however, the closure was unsuccessful.

**Fig. 3:**
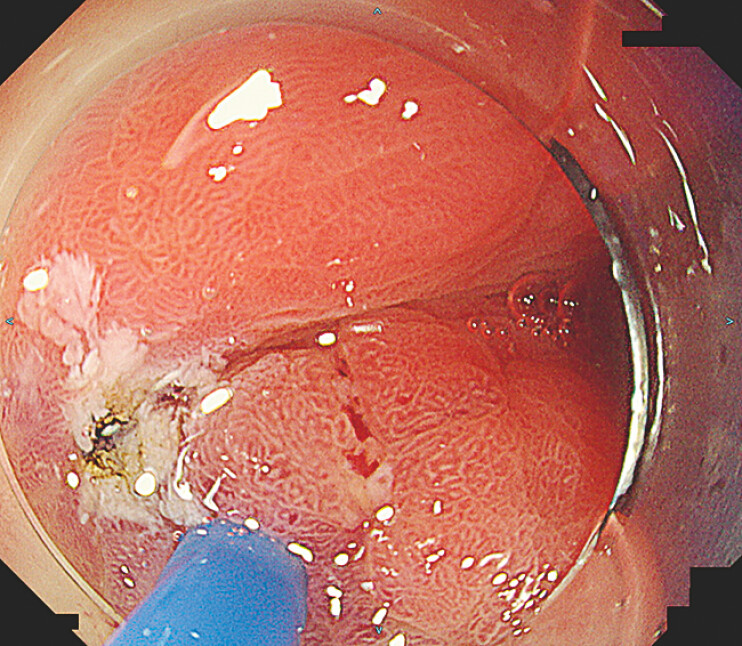
A fresh ulcer bed was created using argon plasma coagulation.

**Fig. 4:**
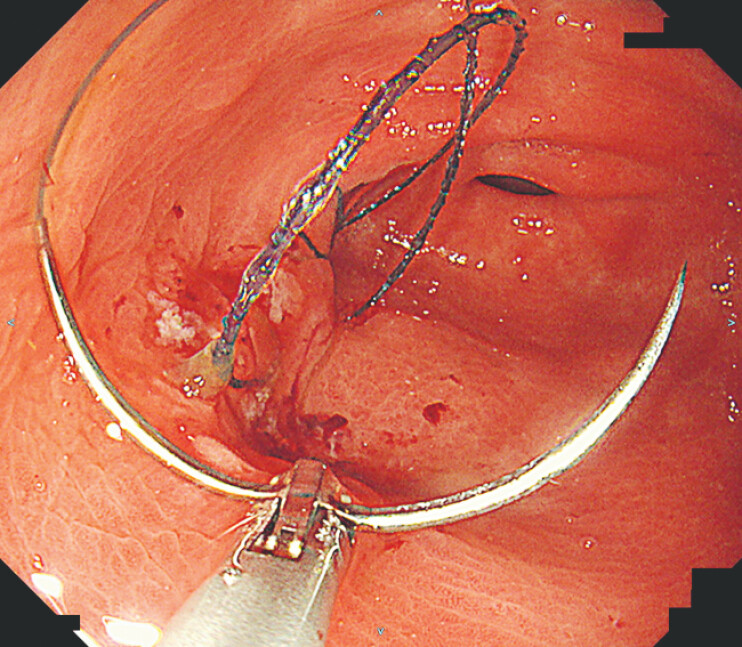
Endoscopic hand suturing was performed to close the fistulous opening.

**Fig. 5:**
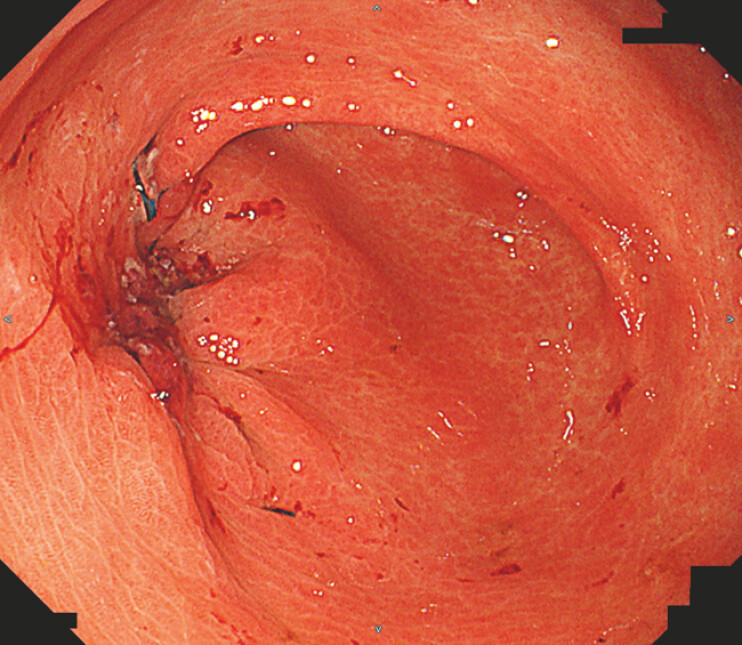
Six sutures were placed, resulting in the complete closure of the fistula.

As the fistulous tract was epithelialized in the endoscopically visible portion of the tract, APC was applied to ablate the mucosa and create a fresh ulcer bed ( Fig. 3 ). EHS using the SutuArt system (Olympus, Co., Ltd, Tokyo, Japan) was subsequently performed ( Fig. 4 ), and six sutures were placed around the fistulous orifice to achieve complete closure ( Fig. 5 ). Follow-up EGD at 1 month confirmed sustained closure ( Video 1 ).

Endoscopic closure of a gastrocutaneous fistula reopening 18 years after gastrostomy removal using argon plasma coagulation and endoscopic hand suturing.Video 1

Although the mechanism of late fistula reopening remains unclear, this case illustrates that a combined endoscopic approach using APC and EHS offers a valuable, minimally invasive option for managing long-standing or recurrent GCF.

Endoscopy_UCTN_Code_TTT_1AO_2AO
